# A sparse autoencoder-based deep neural network for protein solvent accessibility and contact number prediction

**DOI:** 10.1186/s12859-017-1971-7

**Published:** 2017-12-28

**Authors:** Lei Deng, Chao Fan, Zhiwen Zeng

**Affiliations:** 1 School of Software, Central South University, No.22 Shaoshan South Road, Changsha, 410075 China; 2 School of Information Science and Engineering, Central South University, No.932 South Lushan Road, Changsha, 410083 China

**Keywords:** Solvent accessibility, Contact number, Deep neural network, Sequence-derived features

## Abstract

**Background:**

Direct prediction of the three-dimensional (3D) structures of proteins from one-dimensional (1D) sequences is a challenging problem. Significant structural characteristics such as solvent accessibility and contact number are essential for deriving restrains in modeling protein folding and protein 3D structure. Thus, accurately predicting these features is a critical step for 3D protein structure building.

**Results:**

In this study, we present DeepSacon, a computational method that can effectively predict protein solvent accessibility and contact number by using a deep neural network, which is built based on stacked autoencoder and a dropout method. The results demonstrate that our proposed DeepSacon achieves a significant improvement in the prediction quality compared with the state-of-the-art methods. We obtain 0.70 three-state accuracy for solvent accessibility, 0.33 15-state accuracy and 0.74 Pearson Correlation Coefficient (PCC) for the contact number on the 5729 monomeric soluble globular protein dataset. We also evaluate the performance on the CASP11 benchmark dataset, DeepSacon achieves 0.68 three-state accuracy and 0.69 PCC for solvent accessibility and contact number, respectively.

**Conclusions:**

We have shown that DeepSacon can reliably predict solvent accessibility and contact number with stacked sparse autoencoder and a dropout approach.

## Background

Protein 3D structures, determined largely by their amino acid sequences, have been considered as an essential factor for better understanding the function of proteins [1–3]. However, it is exceedingly difficult to directly predict proteins 3D structures from amino acid sequences [4]. Identifying structure properties, such as secondary structure, solvent accessibility or contact number can provide useful insights into the 3D structures [5–7]. Accurate prediction of structural characteristics from the primary sequence is a crucial intermediate step in protein 3D structure prediction [8, 9].

The solvent accessibility (solvent accessible surface area) is defined as the surface region of a residue that is accessible to a rounded solvent while probing the surface of that residue [10]. Solvent burial residues have a particularly strong association with packed amino acids during the folding process [11], and exposed residues give a useful insight into protein-protein interactions and protein stability [12–15]. Information about the degree of surface exposure of an amino acid is commonly used to enhance the understanding of the sequence-structure-function relationship [16, 17]. Besides, it is also helpful to understand a lot of biological problems such as active sites [18], structural epitopes [19, 20], and associations between disease and single nucleotide polymorphisms [21, 22]. In the past, several methods for predicting protein solvent accessible surface area have been implemented mostly in a discrete fashion as the two-state or three-state classification of the exposure rate of residues. Numerous machine learning methods have been applied for solvent exposure prediction based on protein amino acid sequences, including neural networks [5, 23, 24], Bayesian statistic [25], support vector machines [25–27], information theory-based method [28], random forest [29] and nearest neighbor methods [30]. These methods are focused on multistate solvent accessibility prediction, while some other methods attempt to predict the real values of solvent exposure directly [31–33].

In analogous with solvent accessibility, the contact number is another important structural characteristic. The contact number, or coordination number, of a given amino acid, is defined as the number of neighbor residues around the target amino acid within a certain distance. The distances are calculated based on the C-beta atoms. The contact number is also essential for protein structure prediction since the number of possible protein conformations is very limited [34] within a certain number of contacts along the protein chain. During the past few years, there are numerous studies focused on developing computational methods to predict contact number in the protein sequence. Fariselli et al. [35] employed a feed-forward neural network approach with a local window to discriminate between two different states of residue contacts. Kinjo et al. [36] used a simple linear regression scheme based on multiple sequence alignments. Yuan [37] applied SVM to predict two-state and absolute values of contact numbers.

Although the two structure characteristics (solvent accessibility and contact number) are different, they are closely associated with each other representing the structural atmosphere of each residue in the protein structure [36]. Moreover, they may serve as useful restraints for protein folding and tertiary structure prediction. Therefore, developing an integrated computational approach to predict both solvent accessibility and contact number is of great importance.

In this paper, we develop a deep neural network learning-based approach, termed DeepSacon, to significantly improve the prediction performance of both contact number and solvent accessibility by incorporating predicted structure related features and amino acid related features. We pre-train the data with stacked sparse autoencoder, and to prevent units from co-adapting too much. Then, we apply a dropout method in the process of training. The main contributions are as follows: 1) We apply deep learning to better fuse the learned high-level characteristics from protein sequences. 2) Overfitting is significantly reduced and the performance is noticeably improved by combining stacked sparse autoencoder and dropout together. 3) We fully employ specific biological properties such as intrinsic disorder and local backbone angles to further improve the prediction accuracy of contact number and solvent accessibility. We demonstrate that DeepSacon achieves higher performance both in cross-validation and independent test when compared with existing methods.

## Methods

### Datasets

We employ the same training and validation datasets generated in Ma et al.’s [38] for the prediction of solvent accessibility and contact number. Briefly, a monomeric, globular and nonmembrane protein structures of 5729 proteins were obtained from PISCES [39] by removing redundancy (40% cutoff) and length less than 50. This set was randomly divided into a training dataset and a validation dataset of 4583 and 1146 chains, respectively.

In order to further compare with the existing methods, we also collect an independent evaluation dataset of CASP11 proteins. After removing redundant sequences by PISCES (less than 3.0 Å resolution, 0.3 of R-factor and 0.3 cutoff), we obtain a set of 69 proteins out of original CASP11 dataset. In addition, we include the dataset from Yuan’s work [37] as the independent testing dataset to compare with Kinjo’s [36] and Yuan’s methods for contact number prediction.

### Calculation of solvent accessibility

The solvent accessibility (ASA) are computed using the DSSP program [40]. The relative solvent accessibility (RSA) of a residue is calculated as the ratio between the ASA and the maximum solvent accessibility [28]. Based on the RSA value, the classification is classified into three states, that is, buried (B), intermediate (I) and exposed (E). In this study, we use the threshold of 10% for B/I and 40% for I/E for classification of the three-state based on Ma et al.’s work [38].

### Calculation of contact number

The contact number of a residue is defined as the number of other residues located within a sphere of radius *r*_*d*_ centered on the target residue based on the distance between C-beta atoms (C-alpha for glycine). The contact number of the *i*-th residue in a sequence of M residues is calculated by

1 $$ N_{d}^{i}=\!\sum\limits_{j:|j-i|>2}^{M} \!\!\! \sigma (r_{i,j}) \;\;\;\left\{ \begin{array}{ll} \ \sigma (r_{i,j})=1 \ \ if\ r_{i,j}<r_{d} \\ \ \sigma (r_{i,j})=1\ \ if \ r_{i,j}\geq r_{d} \end{array} \right.  $$

where *r*_*i*,*j*_ is the distance between the C-beta atoms of the *i*th and *j*th residues. The cutoff radius *r*_*d*_ is set to 7.5 Å in this work. If the contact number of a residue is above 14, the contact number is set to 14 since such cases are rare in our training data. As a result, a total of 15 states of contact number is calculated for each residue.

### Sequence encoding schemes

For a comprehensive examination, we utilize different sequence-based encoding schemes based on global and local sequence features, which can be grouped into three categories: evolutionary information, predicted structures and amino acid related features. A detailed description of these feature schemes is as follows.

#### Evolutionary information

Previously, evolutionary information has been shown to be useful in structural bioinformatics performance [41, 42]. Position-specific scoring matrix (PSSM) has been widely used for in computational biology [43–48]. In this study, PSSM profiles are calculated with PSI-BLAST against the NCBI nr database (iterations=3 and E-value cutoff=0.001). Also, we compute 20 substitution probabilities from the HMM-profiles produced by HHblits with default parameters against the Uniprot20 database [49]. We scale the values of PSSM and HHM profiles to the range of [0,1] using the following standard logistic function:

2 $$ {x}'=\frac{1}{1+e^{-x}}  $$

where *x* is the raw value and *x* ^′^ is the normalized value of *x*. For a given residue, we have extracted 20+20=40 dimensional vector as evolution related features.

#### Structure related features

Lots of research has shown that local structural characteristics play important roles in predicting solvent accessibility as well as contact number [50–52]. In this paper, we use the predicted secondary structure, predicted natively disordered region and predicted local backbone angles as the structure related features for each position. These three structural features are predicted using the PSIPRED program [53], DISOPRED server [54] and SPIDER2 program [55], respectively. In our previous study, we have shown that using the predicted secondary structure (3 features) and predicted natively disordered region (2 features) could significantly improve the prediction preformation [56]. Some works have also indicated local backbone angles (4 features) have a strong relation with solvent accessibility [55, 57]. We have extracted 3+2+4=9 dimensional vector as structure related features.

#### Amino acid related features

With regard to the global sequence features, the seven physicochemical properties (steric parameter, hydrophobicity, volume, polarizability, isoelectric point, helix probability, sheet probability) of the residues are employed. Besides, we also use contact potential which have proven to be important in the folding of proteins as position independent features [58]. Contact potential has 20 values for each residue. For a given residue, we have extracted a vector of 27 (20+7) dimensions as amino acid related features.

### Prediction method

#### Stacked sparse auto-encoder (SSAE)

**Fig. 1:**
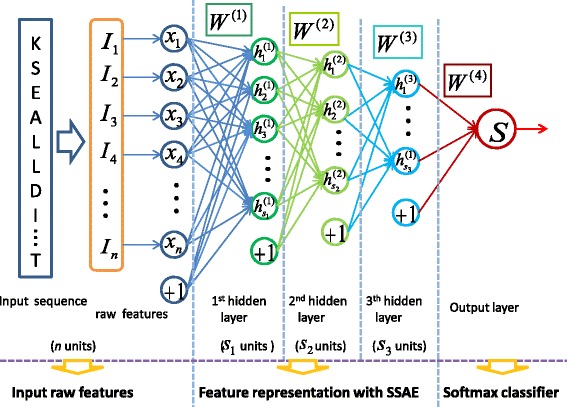
Illustration of Stacked Sparse Autoen-coder (SSAE) by three hidden layers

Stacked auto-encoder (SAE) applies auto-encoder in each layer of a stacked network [59]. We calculate the probability of each label corresponding to each residue based on the given features. Formally, for a target protein with length *L*, we denote the input features as *L*×*N* matrix *X*={*x*_1_,*x*_2_,⋯,*x*_*i*_,⋯,*x*_*L*_},*x*_*i*_∈*R* ^*N*^, where *N* is the number of features for the *i*-th amino acid. The input to the stacked sparse autoencoder (SSAE) is the feature matrix of a protein. Then three hidden SSAE networks are built as shown in Fig. 1, where the sigmoid function is utilized as the activation function. For the input matrix *X*, the goal is to learn and get a feature representation $h_{W,b}=f(W^{T}x)=f\left (\sum _{i=1}^{N}W_{i}x_{i}+b\right)$ at the hidden layer. A conventional auto-encoder would attempt to learn a function *h*_*W*,*b*_≈*x*, which means it is detecting an approximation to the identity function. Here, we add a sparse penalty term to the objective function in the hidden layer to constrain the number of “active” neurons. The mean output value of the hidden layer is kept to 0, which suggests most neurons are supposed to be inactive. The overall cost function of SSAE is defined as:

3 $$ \begin{aligned} J_{sparse}(W,b)=\left [ \frac{1}{N}\sum\limits_{i=1}^{N}\frac{1}{2}\left \| h_{W,b}(x(i))-y(i) \right \|^{2} \right ] \\ + \frac{\lambda }{2}\sum\limits_{l=1}^{n_{l}-1}\sum\limits_{i=1}^{s_{l}}\sum\limits_{j=1}^{s_{l+1}}\left(W_{ji}^{(l)}\right)^{2}+\beta \sum\limits_{j=1}^{s_{2}}KL(\rho \left | \right |\hat{\rho }_{j}) \end{aligned}  $$

where the first part is the term of average sum-of-squares error; *N* is the number of examples in the training set; *λ* is assumed to control the relative weight of the regularization term; *s*_2_ is the number of the hidden neurons; *β* is the weight of the sparsity penalty term; *K* *L*(·) is the Kullback −Leibler divergence [60], which is defined as:

4 $$ KL(\rho \left | \right |\rho_{j})=\rho log\frac{\rho }{\rho_{j}}+(1-\rho)log\frac{1-\rho }{1-\rho_{j}}  $$

The optimal values of the parameter *W* and *b* need to be determined. The two parameters can be computed by minimizing *J*_*sparse*_(*W*,*b*) since the sparse cost function in Eq. (3) is directly associated with the two parameters. This can be implemented using the back-propagation algorithm [61], where the stochastic gradient descent method is applied for training, and the parameters *W* and *b* in each iteration can be updated as:

5 $$ W_{ij}(l)=W_{ij}(l)-\varepsilon \frac{\partial }{\partial W_{ij}(l)}J_{sparse}(W,b)  $$

6 $$ b_{i}(l)=b_{i}(l)-\varepsilon \frac{\partial }{\partial b_{i}(l)}J_{sparse}(W,b)  $$

where *ε* is the learning rate. The back-propagation algorithm works to update the parameters. Finally, for a given amino acid residue *x*, we denote its predicted labels (3-state solvent accessibility or 15-state contact number) as *y*, where *y*∈{1,2,⋯,*M*}, *M*=3 for solvent accessibility and *M*=15 for contact number prediction, the probability of the predicted label *y* is *p*(*y*|*x*;*W*,*b*)=*s* *i* *g* *m* *o* *i* *d*(*W* *x*+*b*).

#### Dropout method

The dropout method can help to reduce “overfitting” when training a neural network with limited data [62]. In this study, we use the dropout approach to build the SSAE to prevent complex co-adaptations and avoid extracting the same features repeatedly. Technically, we can set the output of some hidden neurons to 0 to implement the dropout, since these neurons will not propagate forward in the training process. Note that the dropout in the training and testing process is different, where the dropout is turned off during testing. This will help to promote the feature extraction and prediction performance. Usually, the dropout rate *p* is set to the range from 0.5 to 0.8. We set *p*=0.5 in our experiment.

#### The architecture of our method

**Fig. 2:**
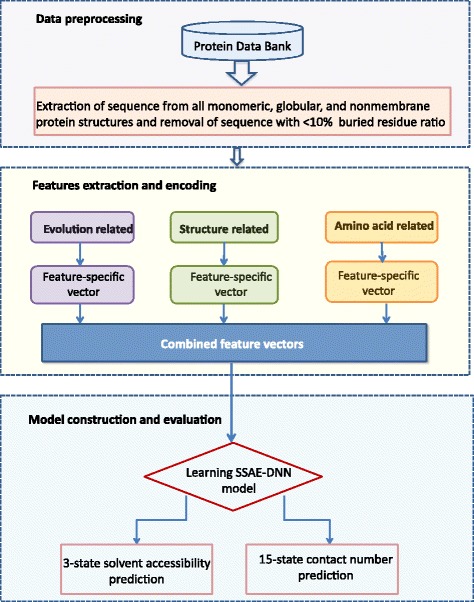
The framework of DeepSacon for residue solvent accessibility and contact number prediction. Three categories of features (evolution, structure, and amino acid) are extracted to build the stacked sparse autoencoder-based deep neural network (SSAE-DNN) model

Figure 2 illustrates the flowchart of the DeepSacon approach which uses a sparse autoencoder-based deep neural network for probing solvent accessibility and contact number from protein primary sequences. In this study, a sliding window method is used to capture the sequence environment. We test a spectrum of window sizes range from 7 to 15 with a step size of 2, and observe that the optimal window size is 11. In our method, a three-layer sparse auot-encoder (SAE) consists of the hidden layers of the deep learning network, and one layer of softmax classifier is added at the top to the output of a 3-state solvent accessibility and a 15-state contact number. The pre-train process with hidden layer sizes of 500, 300, and 150 is implemented by the stochastic gradient descent (SGD) method to tune the weight in the SAE networks. The final deep learning architecture is optimized using the Broyden-Fletcher-Goldfarh-Shanno (BFGS) optimization. Several parameters are fine-tuned using grid search and manual search strategies (sparsity parameter *ρ* = 0.2, weight decay *λ* = 0.003, and weight of the sparsity penalty score *β* = 3).

## Results and discussion

### Performance measures

We calculate accuracy as the primary measure for solvent accessibility as well as contact number. Besides, for the performance evaluation of solvent accessibility, we use precision, recall and *F*_1_-score, defined as follows:

7 $$ Accuracy=\frac{TP+TN}{TP+TN+FP+FN}  $$

8 $$\begin{array}{@{}rcl@{}} Precision&=&\frac{TP}{TP+FP} \end{array} $$

9 $$\begin{array}{@{}rcl@{}} Recall&=&\frac{TP}{TP+FN} \end{array} $$

10 $$\begin{array}{@{}rcl@{}} F_{1}-score&=&\frac{2TP}{2TP+FP+FN} \end{array} $$

where *TP*, *TN*, *FP* and *FN* are the number of the true positive, true negative, false positive and false negative, respectively. For the performance evaluation of contact number, we also compute the Pearson’s correlation coefficient (PCC), defined as the covariance ratio between the predicted and the observed scores.

### Feature importance

**Fig. 3:**
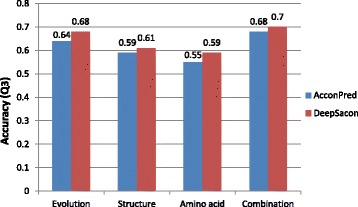
Results of different feature combinations for 3-state solvent accessibility prediction

As mentioned above, we extracted three categories of features, including evolution information, structure features, and amino acid related properties. To evaluate the impact of each feature group on 3-state solvent accessibility prediction, we individually utilize them to build the classifier and perform the prediction. Figure 3 demonstrates the accuracies of different feature groups. From this figure, we can see that using evolution related feature alone could reach 0.68 *Q*_3_ accuracy. Furthermore, we compare the three classes of features respectively with the most recent method AcconPred [38]. We can observe that our method performs significantly better over the AcconPred method.

**Fig. 4:**
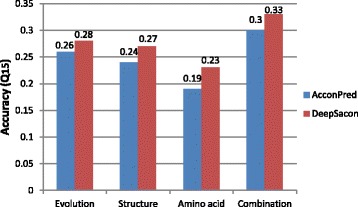
Results of different feature combinations for 15-state contact number prediction

Similarly, we also analyze the relative importance of the three feature groups for predicting contact number. The prediction results of different feature groups and in comparison with AcconPred for 15-state contact number prediction are shown in Fig. 4. We further analyze the variation between the prediction and the observed values. Noted that if this difference is restricted to 2, we could obtain the prediction accuracy of 0.81. We also investigate the prediction performance of our DeepSacon method in terms of PCC, which could reach 0.74.

We further estimate the prediction performance for both solvent accessibility and contact number according to four different feature combinations. We compare the prediction performance on training data with 5-fold cross-validation. As shown in Table 1, we can see that combining all the three feature groups achieve the best performance, which indicates that comprehensive feature encoding schemes can boost the prediction performance.

**Table 1:** Prediction accuracy of 3-state solvent accessibility and 15-state contact number using DeepSacon method based on different feature schemes

Model	Feature	*Q*_3_	*Q*_15_
1	Evolution+Structure	0.678	0.316
2	Evolution+Amino acid	0.687	0.304
3	Structure+Amino acid	0.641	0.286
4	Evolution+Structure+Amino acid	0.7	0.33

We describe detailed results for each label (that is buried, intermediate, and exposed) of solvent accessibility prediction. The three labels are defined with boundaries at 10% and 40% on relative solvent accessibility, and there is an interpretation for such boundaries in Wang’s work. Table 2 gives the all three sates analysis in terms of precision, recall, and *F*1-score. From this table, we observe that the prediction of the buried label is the best, and exposed label is the poorest.

**Table 2:** Performance evaluation for the prediction of 3-state solvent accessibility

Evaluation dataset	Precision	Recall	*F*1-score
Buried	0.79	0.79	0.79
Intermediate	0.71	0.63	0.67
Exposed	0.48	0.65	0.55

### Comparison with other machine learning methods

We compare deep learning with other two broadly used machine learning methods, Support Vector Machine (SVM) and Neural Network, on the training set and CASP11 with 5-fold cross-validation. We implement the algorithms using MATLAB. For SVM, we use RBF as the kernel function. The parameters of C and gamma are set to 1 and 2 respectively based on 5-fold cross-validation. We also evaluate other different kernels and find that RBF performs best. For the neural network, the number of hidden nodes in the first layer is tuned as 300, while in the second layer is 200. The learning rate is set to 0.2. As shown in Table 3, DeepSacon achieves the best performance both on the training set and CASP11. The experiments suggest that deep learning can be successfully applied to the prediction of solvent accessibility and contact number.

**Table 3:** The prediction accuracies of DeepSacon and other machine learning methods in 3-state solvent accessibility and 15-state contact number prediction on the training set and CASP11

	Training set		CASP11	
Method	*Q*_3_	*Q*_15_	*Q*_3_	*Q*_15_
SVM	0.64	0.29	0.61	0.27
NN	0.65	0.28	0.63	0.26
DeepSacon	0.70	0.33	0.68	0.31

### Comparison with other state-of-the-art approaches in independent test

In this section, we compare DeepSacon with other four state-of-the-art solvent accessibility predictors, including a multistep neural-network algorithm by guided weighting scheme (SPINE-X) [63], a nearest neighbor method by using sequence profiles (SANN) [64], an ensemble of Bidirectional Recursive Neural Networks using both sequence and structure similarity (ACCpro5) [65], and a conditional neural fields model (AcconPred) [38]. For contact number prediction, we compare our method with Kinjo’s method which applied linear regression scheme based on multiple sequence alignments [36] and Yuan’s method which employed support vector regression [37]. Table 4 shows the results of these existing methods as well as our method for the 3-state solvent accessibility prediction on the CASP11 dataset. It should be noted that the 3-state outputs of SPINE-X, SANN and Accpro5 are based on different threshold. To objectively compare with our method, we transform the output of these methods uniformly into 3-state at 10%/40% threshold. From Table 4, we find that DeepSacon achieves a significantly better performance over other predictors. It is worth pointing out that the prediction performance improves 2% after using the dropout approach.

**Table 4:** Prediction results of DeepSacon in comparison with other existing methods for 3-state solvent accessibility prediction on CASP11

Method	SPINE-X	SANN	ACCpro5	AcconPred	DeepSacon
*Q*_3_ accuracy	0.57	0.61	0.58	0.64	0.68

We also estimate the probing accuracy and correlation of DeepSacon for 15-state contact number on CASP11. The prediction accuracy is 0.31 for Q15 and is 0.68 for PCC, which agrees with the results on the training dataset (0.33 for Q15 and 0.74 for PCC). Further, we compare our method with Kinjo’s method and Yuan’s on the Yuan dataset. We note that our DeepSacon method exceeds the other approaches significantly. The Pearson correlation coefficient of DeepSacon is 0.69, which exceeds the results of Kinjo’s method (PCC is 0.63) and Yuan’s method (PCC is 0.64).

### Case study

**Fig. 5:**
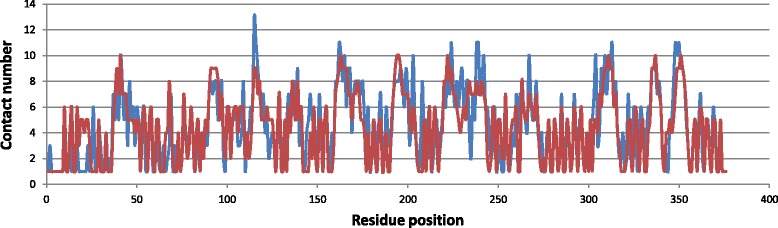
The observed and predicted residue contact number for the HisC protein (PDB entry: 4wbtA). The predicted and observed residue values are colored as blue and red, respectively

**Fig. 6:**
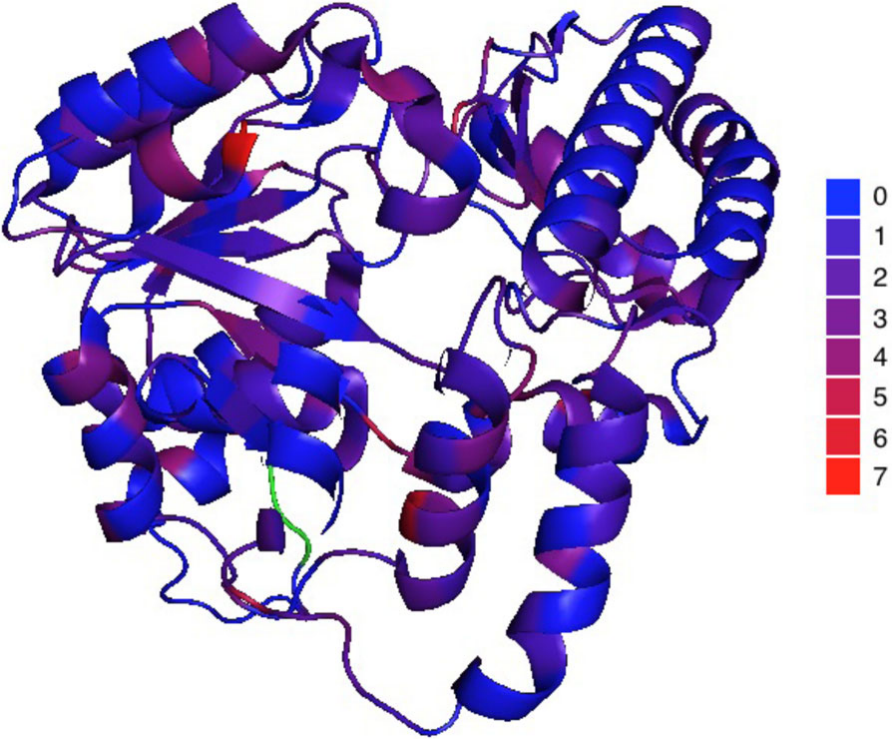
3D visualization of the difference between predicted and observed contact number of the HisC protein (PDB entry: 4wbtA). Different colors represent different numbers of error predicted contact number. The number of errors corresponding to a color is displayed on the right. The closer the blue indicates the more accurate the prediction; otherwise, if it is close to the red, the prediction has more errors

To further demonstrate the prediction capability, we perform a case study by applying DeepSacon to predict the contact number of the histidinol-phosphate aminotransferase protein (HisC, PDBID: 4wbt) with the sequence length of 376 residues from CASP11. The prediction results are shown in Fig. 5. The predicted and observed contact numbers are colored in blue and red, respectively. We can see there is a similar trend between the observed and predicted contact numbers. The predicted and observed values are matched well across most of the protein regions. The PCC value is 0.79, and the mean absolute error (MAE) is 0.46. Figure 6 shows the difference between predicted and observed contact number of each residue of the protein HisC in 3D visualization. We find that the contact number of most residues are well predicted (colored close to blue).

## Conclusions

In this work, we have presented a computational method, DeepSacon, for predicting both solvent accessibility and contact number of proteins by using a deep learning network and employing sequence-derived features, including evolution related features, structure related features, and amino acid related features. The deep learning network is built based on stacked auto-encoder and a dropout method to further improve the performance and reduce the overfitting. DeepSacon provides current state-of-the-art prediction accuracy for solvent accessibility as well as contact number. For solvent accessibility, its *Q*_3_ accuracy reached 0.70 on the 5279 training set and 0.68 on the CASP11 dataset. For contact number, It achieved *Q*_15_ accuracy of 0.33 and 0.31, PCC of 0.74 and 0.68 on training set and CASP11, respectively.

We also compared DeepSacon with traditional machine learning methods such as support vector machines and neural networks. Experimental results indicated DeepSacon has several obvious advantages such as the ability of automatically learned high-level features and stronger generalization capability.

Actually, accurate homology structure information is of crucial importance to structural characteristics prediction. Unfortunately, the number of proteins with completely homology structure information is far less than that with unknown homology structure information. Since DeepSacon can predict the solvent accessibility and contact number from simple primary sequences in the absence of protein structures, it has more extensive applications. Moreover, our work provides a complementary and useful approach towards the more accurate prediction of other structural properties.

## References

[1] Baker D, Sali A (2001). Protein structure prediction and structural genomics. Science.

[2] Wei L, Zou Q (2016). Recent progress in machine learning-based methods for protein fold recognition. Int J Mol Sci.

[3] Zhang Z, Zhang J, Fan C, Tang Y, Deng L. Katzlgo: Large-scale prediction of lncrna functions by using the katz measure based on multiple networks. IEEE/ACM Trans Comput Biol Bioinforma. 2017. doi:10.1109/TCBB.2017.2704587. 10.1109/TCBB.2017.2704587 28534780

[4] Dill KA, MacCallum JL (2012). The protein-folding problem, 50 years on. Science.

[5] Pollastri G, Baldi P, Fariselli P, Casadio R (2002). Prediction of coordination number and relative solvent accessibility in proteins. Proteins Struct Funct Bioinforma.

[6] Adamczak R, Porollo A, Meller J (2005). Combining prediction of secondary structure and solvent accessibility in proteins. Proteins Struct Funct Bioinforma.

[7] Wei L, Liao M, Gao X, Zou Q (2015). An improved protein structural classes prediction method by incorporating both sequence and structure information. IEEE Trans Nanobioscience.

[8] Bowie JU, Luthy R, Eisenberg D (1991). A method to identify protein sequences that fold into a known three-dimensional structure. Science.

[9] Rost B, Sander C (1994). Conservation and prediction of solvent accessibility in protein families. Proteins Struct Funct Bioinforma.

[10] Lee B, Richards FM (1971). The interpretation of protein structures: estimation of static accessibility. J Mol Biol.

[11] Hartl FU, Bracher A, Hayer-Hartl M (2011). Molecular chaperones in protein folding and proteostasis. Nature.

[12] Ma B, Elkayam T, Wolfson H, Nussinov R (2003). Protein–protein interactions: structurally conserved residues distinguish between binding sites and exposed protein surfaces. Proc Natl Acad Sci.

[13] Khashan R, Zheng W, Tropsha A (2012). Scoring protein interaction decoys using exposed residues (spider): a novel multibody interaction scoring function based on frequent geometric patterns of interfacial residues. Proteins Struct Funct Bioinforma.

[14] Liu H, Sun J, Guan J, Zheng J, Zhou S (2015). Improving compound-protein interaction prediction by building up highly credible negative samples. Bioinformatics.

[15] Garzón JI, Deng L, Murray D, Shapira S, Petrey D, Honig B (2016). A computational interactome and functional annotation for the human proteome. Elife.

[16] Eyal E, Najmanovich R, Mcconkey BJ, Edelman M, Sobolev V (2004). Importance of solvent accessibility and contact surfaces in modeling side-chain conformations in proteins. J Comput Chem.

[17] Totrov M (2004). Accurate and efficient generalized born model based on solvent accessibility: derivation and application for logp octanol/water prediction and flexible peptide docking. J Comput Chem.

[18] Huang B, Schroeder M (2006). Ligsite csc: predicting ligand binding sites using the connolly surface and degree of conservation. BMC Struct Biol.

[19] Haste Andersen P, Nielsen M, Lund O (2006). Prediction of residues in discontinuous b-cell epitopes using protein 3d structures. Protein Sci.

[20] Wei L, Xing P, Tang J, Zou Q (2017). Phospred-rf: a novel sequence-based predictor for phosphorylation sites using sequential information only. IEEE Trans NanoBioscience.

[21] Mooney S (2005). Bioinformatics approaches and resources for single nucleotide polymorphism functional analysis. Brief Bioinforma.

[22] Zhang J, Zhang Z, Chen Z, Deng L. Integrating multiple heterogeneous networks for novel lncrna-disease association inference. IEEE/ACM Trans Comput Biol Bioinforma. 2017. doi:10.1109/TCBB.2017.2701379. 10.1109/TCBB.2017.2701379 28489543

[23] Ahmad S, Gromiha MM (2002). Netasa: neural network based prediction of solvent accessibility. Bioinformatics.

[24] Adamczak R, Porollo A, Meller J (2004). Accurate prediction of solvent accessibility using neural networks–based regression. Proteins Struct Funct Bioinforma.

[25] Thompson MJ, Goldstein RA (1996). Predicting solvent accessibility: Higher accuracy using bayesian statistics and optimized residue substitution classes. Proteins.

[26] Kim H, Park H (2004). Prediction of protein relative solvent accessibility with support vector machines and long-range interaction 3d local descriptor. Proteins Struct Funct Bioinforma.

[27] Nguyen MN, Rajapakse JC (2005). Prediction of protein relative solvent accessibility with a two-stage svm approach. Proteins Struct Funct Bioinforma.

[28] Naderi-Manesh H, Sadeghi M, Arab S, Moosavi Movahedi AA (2001). Prediction of protein surface accessibility with information theory. Proteins Struct Funct Bioinforma.

[29] Pugalenthi G, Kumar Kandaswamy K, Chou KC, Vivekanandan S, Kolatkar P (2012). Rsarf: prediction of residue solvent accessibility from protein sequence using random forest method. Protein Pept Lett.

[30] Sim J, Kim SY, Lee J (2005). Prediction of protein solvent accessibility using fuzzy k-nearest neighbor method. Bioinformatics.

[31] Chang DT-H, Huang HY, Syu YT, Wu CP (2008). Real value prediction of protein solvent accessibility using enhanced pssm features. BMC Bioinformatics.

[32] Zhang J, Chen W, Sun P, Zhao X, Ma Z (2015). Prediction of protein solvent accessibility using pso-svr with multiple sequence-derived features and weighted sliding window scheme. BioData Min.

[33] Nguyen MN, Rajapakse JC (2006). Two-stage support vector regression approach for predicting accessible surface areas of amino acids. Proteins Struct Funct Bioinforma.

[34] Kabakcioglu A, Kanter I, Vendruscolo M, Domany E (2001). Statistical properties of contact vectors. Phys Rev E.

[35] Fariselli P, Casadio R (2000). Prediction of the number of residue contacts in proteins. Proceedings of the 8th International Conference on Intelligent Systems for Molecular Biology (ISMB 2000), vol 8.

[36] Kinjo AR, Horimoto K, Nishikawa K (2005). Predicting absolute contact numbers of native protein structure from amino acid sequence. Proteins Struct Funct Bioinforma.

[37] Yuan Z (2005). Better prediction of protein contact number using a support vector regression analysis of amino acid sequence. BMC Bioinformatics.

[38] Ma J, Wang S (2015). Acconpred: Predicting solvent accessibility and contact number simultaneously by a multitask learning framework under the conditional neural fields model. BioMed Res Int.

[39] Wang G, Jr DR (2003). Pisces: a protein sequence culling server. Bioinformatics.

[40] Kabsch W, Sander C (1983). Dictionary of protein secondary structure: pattern recognition of hydrogen-bonded and geometrical features. Biopolymers.

[41] Biasini M, Bienert S, Waterhouse A, Arnold K, Studer G, Schmidt T, Kiefer F, Cassarino TG, Bertoni M, Bordoli L (2014). Swiss-model: modelling protein tertiary and quaternary structure using evolutionary information. Nucleic Acids Res.

[42] Ramsey DC, Scherrer MP, Zhou T, Wilke CO (2011). The relationship between relative solvent accessibility and evolutionary rate in protein evolution. Genetics.

[43] Zhang J, Zhao X, Sun P, Ma Z (2013). Psno: predicting cysteine s-nitrosylation sites by incorporating various sequence-derived features into the general form of chou’s pseaac. Int J Mol Sci.

[44] Song J, Burrage K, Zheng Y, Huber T (2006). Prediction of cis/trans isomerization in proteins using psi-blast profiles and secondary structure information. BMC Bioinformatics.

[45] Chen K, Kurgan L (2007). Pfres: protein fold classification by using evolutionary information and predicted secondary structure. Bioinformatics.

[46] Zou Q, Wan S, Ju Y, Tang J, Zeng X (2016). Pretata: predicting tata binding proteins with novel features and dimensionality reduction strategy. BMC Syst Biol.

[47] Zou Q, Zeng J, Cao L, Ji R (2016). A novel features ranking metric with application to scalable visual and bioinformatics data classification. Neurocomputing.

[48] Gan Y, Tao H, Zou G, Yan C, Guan J (2016). Dynamic epigenetic mode analysis using spatial temporal clustering. BMC Bioinformatics.

[49] Remmert M, Biegert A, Hauser A, Söding J (2012). Hhblits: lightning-fast iterative protein sequence searching by hmm-hmm alignment. Nat Methods.

[50] Dyson HJ, Wright PE (2005). Intrinsically unstructured proteins and their functions. Nat Rev Mol Cell Biol.

[51] Haynes C, Oldfield CJ, Ji F, Klitgord N, Cusick ME, Radivojac P, Uversky VN, Vidal M, Iakoucheva LM (2006). Intrinsic disorder is a common feature of hub proteins from four eukaryotic interactomes. PLoS Comput Biol.

[52] Yang Y, Heffernan R, Paliwal K, Lyons J, Dehzangi A, Sharma A, Wang J, Sattar A, Zhou Y (2017). Spider2: A package to predict secondary structure, accessible surface area, and main-chain torsional angles by deep neural networks. Methods Mol Biol.

[53] Jones DT (1999). Protein secondary structure prediction based on position-specific scoring matrices. J Mol Biol.

[54] Ward JJ, Sodhi JS, McGuffin LJ, Buxton BF, Jones DT (2004). Prediction and functional analysis of native disorder in proteins from the three kingdoms of life. J Mol Biol.

[55] Heffernan R, Paliwal K, Lyons J, Dehzangi A, Sharma A, Wang J, Sattar A, Yang Y, Zhou Y (2015). Improving prediction of secondary structure, local backbone angles, and solvent accessible surface area of proteins by iterative deep learning. Sci Rep..

[56] Fan C, Liu D, Huang R, Chen Z, Deng L (2016). Predrsa: a gradient boosted regression trees approach for predicting protein solvent accessibility. BMC Bioinformatics.

[57] Lyons J, Dehzangi A, Heffernan R, Sharma A, Paliwal K, Sattar A, Zhou Y, Yang Y (2014). Predicting backbone c *α* angles and dihedrals from protein sequences by stacked sparse auto-encoder deep neural network. J Comput Chem.

[58] Betancourt MR, Thirumalai D (1999). Pair potentials for protein folding: choice of reference states and sensitivity of predicted native states to variations in the interaction schemes. Protein Sci.

[59] Bengio Y, Lamblin P, Popovici D, Larochelle H (2007). Greedy layer-wise training of deep networks. Adv Neural Inf Process Syst.

[60] Kullback S, Leibler RA (1951). On information and sufficiency. Ann Math Stat.

[61] Rumelhart DE, Hinton GE, Williams RJ (1988). Learning representations by back-propagating errors. Cogn Model.

[62] Hinton GE, Srivastava N, Krizhevsky A, Sutskever I, Salakhutdinov RR (2012). Improving neural networks by preventing co-adaptation of feature detectors. Comput Sci.

[63] Faraggi E, Xue B, Zhou Y (2009). Improving the prediction accuracy of residue solvent accessibility and real-value backbone torsion angles of proteins by guided-learning through a two-layer neural network. Proteins Struct Funct Bioinforma.

[64] Joo K, Lee SJ, Lee J (2012). Sann: solvent accessibility prediction of proteins by nearest neighbor method. Proteins Struct Funct Bioinforma.

[65] Magnan CN, Baldi P (2014). Sspro/accpro 5: almost perfect prediction of protein secondary structure and relative solvent accessibility using profiles, machine learning and structural similarity. Bioinformatics.

